# Supplementary material for: Determinants of non-adherence to home injury prevention practice among parents of under-five children in North Seberang Perai district, Penang: A mixed-methods study protocol
Source: PLoS One. 2023 Aug 16;18(8):e0282995. doi: 10.1371/journal.pone.0282995 (PMC10431611; doi:10.1371/journal.pone.0282995)
Supplement: S1 Appendix — (DOCX) [file pone.0282995.s001.docx]

**APPENDIX 1: INTERVIEW GUIDE**

**Preamble**

Introduce myself and my affiliation.

Explain the purpose of the interview.

Participant

- What is your name? What should I call you?
- How old are you?
- What do you do?

Participants home

- Where do you live?
- How long have you lived there?
- What type of house?
- Is it owned or rented?

Participant’s family

- What does your husband/wife do?
- Who else lives with you?
- How many children do you have?
- How old are they?
- Do you send them to nursery?

Participant’s activities

- What is your daily activities?
- How do you travel?

**Research questions**

|  | **Variable** | **Preamble** | **Questions** |
| --- | --- | --- | --- |
| **1** | **Home injury prevention practice** | Have your child ever sustain any injury at home? | - Why do you think it happened? - On your opinion, how can we prevent this from happening?   1. Did you do all the things you just said?   2. If yes, why it still happened?   3. If not, why? |
| **2** | **Parental perceived susceptibility** | Is it frequent that your child get injured at home? | - Yes/no, why? - What type of injury? - How does it happened? |
| **3** | **Parental perceived severity** | You said your child had sustained injury before right?  Or  Did you know of a family members or friends whose child sustained an injury? | - What type & seriousness? When? Where? How? Why? (or just: what happened?)   How did it affect you?   - Probe: physical, social, financial - Probe: Short term & long term   How did it affect the child?   - Probe: physical, social, financial - Probe: Short term & long term |
| **4** | **Parental perceived benefit** | Do you think that preventive measures beneficial to your children? | - If yes, what are the benefit? – example of injury and the prevention measures - If not, why? - How effective the measures in benefiting your child? - How do you think the benefit compares with the cost? |
| **5** | **Parental perceived barrier** | Do you think it is difficult to prevent home injury from your children? | What are the barriers?   - Time - Money - Support (family, community, government) - Distraction (other child, gadgets, etc)   Do you think the barrier can be overcome?  How do you overcome the barriers? |
| **6** | **Parental self-efficacy** | How confident are you in preventing home injury? | - Do you think you have the information on child injury prevention? - Where and how did you get the information? - How the info helped you in preventing injury at home? - What do you need to help you improve your confidence? (example: subsidy, training) |
| **7** | **Perceived social norm** | Is it normal to get injury at home? | - Yes/no, why? - Probe regarding culture, community norm   Is home injury prevention practice a norm? A must? Or is it rare?   - Probe: culture, local community norm   What are usually/normally used as prevention measures at home?   - Probe: fall, drowning, poisoning, suffocation, burn, RTA |
| **8** | **Parental attitude** | What do you think about preventing home injury? | - Is it a parent’s responsibility? Mother or father or both? - Does anyone else has a role in providing safety environment for children at home? Probe: house developer, doctors/nurses |
| **9** | **Knowledge** | How much you ever heard or learn about home injury prevention? | - From where? - Is it on your own initiative or from accidental encounter or from an advocator? - Is it effective in giving you awareness and new knowledge? - Where do you think you can go to get more information? - What is your preference in receiving info regarding this? |
| **10** | **Type of house and house hazards** | What is the type of the house you are currently living?  What is your opinion regarding your house with regards to injury? | Do you consider your house safe for children? Why?  What do you think about the building and its surroundings?   - Flats: risk of fall? - Terrace: are there gates/barrier outside the house? - Are there uncovered drain or hole?   What do you think about the house design?   - Windows easily opened? - Low electrical sockets - Can the toilet door locked from outside?   What do you think of your furniture?  Are there any hazards at your home?   - Probe: fall, burn (smoking), choking/suffocation, drowning, poisoning and road traffic accident |
| **11** | **Parent age** | You said your age is …, | Do you think your age affects your prevention measures at home? Why? How? |
| **12** | **Parent gender** | Being a male/female, | Do you think male or female is better at home injury prevention? Why? How? |
| **13** | **Ethnicity** | Being … ethnicity, | Does that influence your prevention? Why? |
| **14** | **Parent education level** | What is the last education you received? | Does that influence your safety measures at home? Why? How? |
| **15** | **Household income** | So your household generate about … per month. | Does it affect your prevention measures? How? |
| **16** | **Marital status** | Being married/ unmarried/divorced.. | How does it affect your prevention measures at home? |
| **17** | **Number of children** | How many children do you have? | How does it affect your safety measures?  Experience? |
| **18** | **Child age** | How old is your child? | Does it influence you to take precautions? Why?  Which age is the most vulnerable? Why? |
| **19** | **Child gender** | Is your child boy or girl? | Does it influence you to take precautions? Why?  Which gender is more concerning? Why? |
| **20** | **Child temperament** | How does your child react to his/her surroundings or to people? | Does that effect your safety measures? Why? How? |
| **21** | **Child injury history** | You said you child had/never had injury at home? | How does that influence your preventive measures?  Why? |

**Conclusion**

Is there anything you want to add or clarify?

Is there anything you want to ask?

I would like to meet you again for any clarification regarding the interview.

Thank you for your time and sharing.
