# Supplementary material for: Determinants of non-adherence to home injury prevention practice among parents of under-five children in North Seberang Perai district, Penang: A mixed-methods study protocol
Source: PLoS One. 2023 Aug 16;18(8):e0282995. doi: 10.1371/journal.pone.0282995 (PMC10431611; doi:10.1371/journal.pone.0282995)
Supplement: S1 File — (DOCX) [file pone.0282995.s002.docx]

**Questionnaire – English Version**

| **Code no.** |  |  |  |  |
| --- | --- | --- | --- | --- |


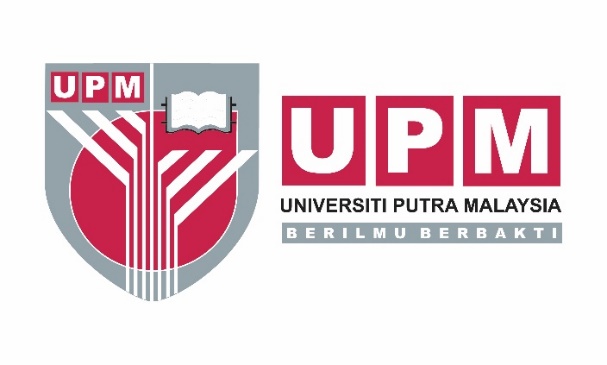


**Questionnaire**

**Determinants of Non-adherence to Unintentional Home Injury Prevention Practice among Parents of Under-five Children in North Seberang Perai District, Penang**

**Researcher:**

**Dr Nurul Iman binti Abdul Rahim**

**Doctor of Public Health Student UPM**

**Supervisor:**

**Associate Prof Dr Hayati binti Kadir @ Shahar**

**Senior Lecturer Department of Community Health UPM**

**Co-supervisors:**

**Associate Prof Dr Nor Afiah Mohd Zulkefli**

**Senior Lecturer Department of Community Health UPM**

**Dr Ahmad Iqmer Nashriq bin Mohd Nazan**

**Lecturer Department of Community Health UPM**

This questionnaire has 19 pages with 9 sections as below:

| A | Sociodemographic status of parent and child |
| --- | --- |
| B | Child temperament |
| C | Parenting style |
| D | Perception towards home injury prevention |
| E | Social norm |
| F | Parent’s attitude towards home injury prevention |
| G | Parent’s knowledge on child home safety |
| H | Parent’s home injury prevention practice |
| I | House hazards |

1. **SOCIODEMOGRAPHIC STATUS OF PARENT & CHILD**

| Instruction: Please answer ALL the questions. Please tick (√) at only ONE relevant box for each question. Please answer the questions regarding your child based on your youngest child. | |
| --- | --- |
| **No.** | **Item** |
| A1. | Age: _________ years |
| A2. | Sex: Male Female  v  v |
| A3. | Ethnicity: Malay Chinese Indian Others, please state ______________  v  v  v  v |
| A4. | Education level: No formal Primary  v  v  Secondary University/college  v  v |
| A5. | Household monthly income (estimate): RM______________ |
| A6. | Employment status: Employed Unemployed Self-employed  v  v  v |
| A7. | Marital status: Single Married Widower/divorce  v  v  v |
| A8 | Number of child: ___________ child/children |
| A9. | Type of house you currently lived in:  Terrace/cluster/semi-D single-storey  v  Terrace/cluster/semi-D double-storey or higher  v  Apartment/condominium  v  Flats, please state house level _________  v  Bungalow single storey  v  Bungalow double storey or higher  v  Others, please state _________________________  v |
| A10. | Child’s date of birth: _____/ _____/ ________ (date/month/year) |
| A11. | Child’s sex: Male Female  v  v |
| A12. | In the past one year, does your child ever sustain injury at home?  Yes (continue to question A13)  v  No (no need to answer A13, continue to section B)  v |
| A13. | What type of injury did your child sustained (you can answer more than one)?  Fall Drowning  v  v  Burn/scalds Road traffic accident (being hit by any type  v  v  Choking/suffocate of vehicle at your home surrounding)  v  Poisoning Animal bite  v  v |

**B. CHILD TEMPERAMENT**

Direction: For this section, please answer ONLY ONE SET fo questions based on your child’s age. Set I is for age 3 to 12 months, Set II is for 13 to 36 months and Set III is for age 37 months and above.

**Set I: Age 3 to 12 months**

| As you read each description of the baby’s behaviour below, please indicate how often the baby did this during the LAST WEEK (the past seven days) by ticking (√)  on one of the numbers in the right column. These numbers indicate how often you observed the behaviour described during the last week:  **1: Never 2: Very rarely 3: Less than half the time 4: About half the time 5: More than half the time 6: Almost always 7: Always**  The “Not applicable” column is used when you did not see the baby in the situation described during the last week. For example, if the situation mentions the baby having to wait for food or liquids and there was no time during the last week when the baby had to wait, tick (√) at NA box. “Not applicable” is different from “Never” (1). “Never” is used when you saw the baby in the situation but the baby never engaged in the behaviour listed during the last week. For example, if the baby did have to wait for food or  liquids at least once but never cried loudly while waiting, tick (√) at the (1) column. Please be sure to tick (√) a number or NA for every item. | | | | | | | | | |
| --- | --- | --- | --- | --- | --- | --- | --- | --- | --- |
| **No** | **Statement** | **1** | **2** | **3** | **4** | **5** | **6** | **7** | **NA** |
| B1. | When being dressed or undressed during the last week, how often did the baby squirm and/or try to roll away? |  |  |  |  |  |  |  |  |
| B2. | When tossed around playfully how often did the baby laugh? |  |  |  |  |  |  |  |  |
| B3. | When tired, how often did your baby show distress? |  |  |  |  |  |  |  |  |
| B4. | When introduced to an unfamiliar adult, how often did the baby cling to a parent? |  |  |  |  |  |  |  |  |
| B5. | How often during the last week did the baby enjoy being read to? |  |  |  |  |  |  |  |  |
| B6. | How often during the last week did the baby play with one toy or object for 5-10 minutes? |  |  |  |  |  |  |  |  |
| B7. | How often during the week did your baby move quickly toward new objects? |  |  |  |  |  |  |  |  |
| B8. | When put into the bath water, how often did the baby laugh? |  |  |  |  |  |  |  |  |
| B9. | When it was time for bed or a nap and your baby did not want to go, how often did s/he whimper or sob? |  |  |  |  |  |  |  |  |
| B10. | After sleeping, how often did the baby cry if someone doesn’t come within a few minutes? |  |  |  |  |  |  |  |  |
| B11. | In the last week, while being fed in your lap, how often did the baby seem eager to get away as soon as the feeding was over? |  |  |  |  |  |  |  |  |
| B12. | When singing or talking to your baby, how often did s/he soothe immediately? |  |  |  |  |  |  |  |  |
| B13. | When placed on his/her back, how often did the baby squirm and/or turn body? |  |  |  |  |  |  |  |  |
| B14. | During a peekaboo game, how often did the baby laugh? |  |  |  |  |  |  |  |  |
| B15. | How often does the infant look up from playing when the telephone rings? |  |  |  |  |  |  |  |  |
| B16. | How often did the baby seem angry (crying and fussing) when you left her/him in the crib? |  |  |  |  |  |  |  |  |
| B17. | How often during the last week did the baby startle at a sudden change in body position (e.g., when moved suddenly)? |  |  |  |  |  |  |  |  |
| B18. | How often during the last week did the baby enjoy hearing the sound of words, as in nursery rhymes? |  |  |  |  |  |  |  |  |
| B19. | How often during the last week did the baby look at pictures in books and/or magazines for 5 minutes or longer at a time? |  |  |  |  |  |  |  |  |
| B20. | When visiting a new place, how often did your baby get excited about exploring new surroundings? |  |  |  |  |  |  |  |  |
| B21. | How often during the last week did the baby smile or laugh when given a toy? |  |  |  |  |  |  |  |  |
| B22. | At the end of an exciting day, how often did your baby become tearful? |  |  |  |  |  |  |  |  |
| B23. | How often during the last week did the baby protest being placed in a confining place (infant seat, play pen, car seat, etc.)? |  |  |  |  |  |  |  |  |
| B24. | When being held, in the last week, did your baby seem to enjoy him/herself? |  |  |  |  |  |  |  |  |
| B25. | When being held, in the last week, did your baby seem to enjoy him/herself? |  |  |  |  |  |  |  |  |
| B26. | When hair was washed, how often did the baby vocalize? |  |  |  |  |  |  |  |  |
| B27. | How often did your baby notice the sound of an airplane passing overhead? |  |  |  |  |  |  |  |  |
| B28. | When introduced to an unfamiliar adult, how often did the baby refuse to go to the unfamiliar person? |  |  |  |  |  |  |  |  |
| B29. | When you were busy with another activity, and your baby was not able to get your attention, how often did s/he cry? |  |  |  |  |  |  |  |  |
| B30. | How often during the last week did the baby enjoy gentle rhythmic activities, such as rocking or swaying? |  |  |  |  |  |  |  |  |
| B31. | How often during the last week did the baby stare at a mobile, crib bumper or picture for 5 minutes or longer? |  |  |  |  |  |  |  |  |
| B32. | When the baby wanted something, how often did s/he become upset when s/he could not get what s/he wanted? |  |  |  |  |  |  |  |  |
| B33. | When in the presence of several unfamiliar adults, how often did the baby cling to a parent? |  |  |  |  |  |  |  |  |
| B34. | When rocked or hugged, in the last week, did your baby seem to enjoy him/herself? |  |  |  |  |  |  |  |  |
| B35. | When patting or gently rubbing some part of the baby’s body, how often did s/he soothe immediately? |  |  |  |  |  |  |  |  |
| B36. | How often did your baby make talking sounds when riding in a car? |  |  |  |  |  |  |  |  |
| B37. | When placed in an infant seat or car seat, how often did the baby squirm and turn body? |  |  |  |  |  |  |  |  |

**Set II: Age 13 to 36 months**

| As you read each description of the child’s behaviour below, please indicate how often the child did this during the last TWO WEEKS by ticking (√) at one of the numbers at the right column. These numbers indicate how often you observed the behaviour described during the last two weeks:  **1: Never 2: Very rarely 3: Less than half the time 4: About half the time 5: More than half the time 6: Almost always 7: Always**  The “Not Applicable” column (NA) is used when you did not see the child in the situation described during the last two weeks. For example, if the situation mentions the child going to the doctor and there was no time during the last two weeks when the child went to the doctor, tick (√) at the (NA) column. “Not applicable” (NA) is different from “Never” (1). “Never” is used when you saw the child in the situation but the child never engaged in the behaviour mentioned in the last two weeks. Please be sure to tick (√) a number or NA for every item. | | | | | | | | | |
| --- | --- | --- | --- | --- | --- | --- | --- | --- | --- |
| **No** | **Statement** | **1** | **2** | **3** | **4** | **5** | **6** | **7** | **NA** |
| B1. | When approached by an unfamiliar person in a public place (for example, the grocery store), how often did your child cling to a parent? |  |  |  |  |  |  |  |  |
| B2. | While having trouble completing a task (e.g., building, drawing, dressing), how often did your child get easily irritated? |  |  |  |  |  |  |  |  |
| B3. | When a familiar child came to your home, how often did your child seek out the company of the child? |  |  |  |  |  |  |  |  |
| B4. | When offered a choice of activities, how often did your child decide what to do very quickly and go after it? |  |  |  |  |  |  |  |  |
| B5. | During daily or evening quiet time with you and your child, how often did your child enjoy just being quietly sung to? |  |  |  |  |  |  |  |  |
| B6. | While playing outdoors, how often did your child choose to take chances for the fun and excitement of it? |  |  |  |  |  |  |  |  |
| B7. | When engaged in play with his/her favourite toy, how often did your child play for more than 10 minutes? |  |  |  |  |  |  |  |  |
| B8. | When engaged in play with his/her favourite toy, how often did your child continue to play while at the same time responding to your remarks or questions? |  |  |  |  |  |  |  |  |
| B9. | When told that loved adults would visit, how often did your child get very excited? |  |  |  |  |  |  |  |  |
| B10. | During quiet activities, such as reading a story, how often did your child fiddle with his/her hair, clothing, etc.? |  |  |  |  |  |  |  |  |
| B11. | While playing indoors, how often did your child like rough and rowdy games? |  |  |  |  |  |  |  |  |
| B12. | When being gently rocked or hugged, how often did your child seem eager to get away? |  |  |  |  |  |  |  |  |
| B13. | When encountering a new activity, how often did your child get involved immediately? |  |  |  |  |  |  |  |  |
| B14. | When engaged in an activity requiring attention, such as building with blocks, how often did your child tire of the activity relatively quickly? |  |  |  |  |  |  |  |  |
| B15. | During everyday activities, how often did your child pay attention to you right away when you called to him/her? |  |  |  |  |  |  |  |  |
| B16. | During everyday activities, how often did your child seem to be irritated by tags in his/her clothes? |  |  |  |  |  |  |  |  |
| B17. | During everyday activities, how often did your child become bothered by sounds while in noisy environments? |  |  |  |  |  |  |  |  |
| B18. | During everyday activities, how often did your child seem full of energy, even in the evening? |  |  |  |  |  |  |  |  |
| B19. | While in a public place, how often did your child seem afraid of large, noisy vehicles? |  |  |  |  |  |  |  |  |
| B20. | When playing outdoors with other children, how often did your child seem to be one of the most active children? |  |  |  |  |  |  |  |  |
| B21. | When told “no”, how often did your child stop the forbidden activity? |  |  |  |  |  |  |  |  |
| B22. | When told “no”, how often did your child become sadly tearful? |  |  |  |  |  |  |  |  |
| B23. | Following an exciting activity or event, how often did your child seem to feel down or blue? |  |  |  |  |  |  |  |  |
| B24. | While playing indoors, how often did your child run through the house? |  |  |  |  |  |  |  |  |
| B25. | Before an exciting event (such as receiving a new toy), how often did your child get very excited about getting it? |  |  |  |  |  |  |  |  |
| B.26 | When s/he asked for something and you said “no”, how often did your child have a temper tantrum? |  |  |  |  |  |  |  |  |
| B27. | When asked to wait for a desirable item (such as ice cream), how often did your child wait patiently? |  |  |  |  |  |  |  |  |
| B28. | When being gently rocked, how often did your child smile? |  |  |  |  |  |  |  |  |
| B29. | While being held on your lap, how often did your child mold to your body? |  |  |  |  |  |  |  |  |
| B30. | When a familiar adult, such as a relative or friend, visited your home, how often did your child want to interact with the adult? |  |  |  |  |  |  |  |  |
| B31. | When asked to do so, how often was your child able to be careful with something breakable? |  |  |  |  |  |  |  |  |
| B32. | When visiting a new place, how often did your child not want to enter? |  |  |  |  |  |  |  |  |
| B33. | When s/he was upset, how often did your child cry for more than 3 minutes, even when being comforted? |  |  |  |  |  |  |  |  |
| B34. | When s/he was upset, how often did your child become easily soothed? |  |  |  |  |  |  |  |  |
| B35. | When you were busy, how often did your child find another activity to do when asked? |  |  |  |  |  |  |  |  |
| B36. | When around large gatherings of familiar adults or children, how often did your child enjoy playing with a number of different people? |  |  |  |  |  |  |  |  |

**Set III: Age 37 months and above**

| Instruction: We would like you to tell us what your child's reaction is likely to be in those situations. There are of course no "correct" ways of reacting; children differ widely in their reactions, and it is these differences we are trying to learn about. Please read each statement and decide whether it is a "true" or "untrue" description of your child's reaction within the past six months. Use the following scale to indicate how well a statement describes your child:  **1: Extremely untrue 2: Quite untrue 3: Slightly untrue 4: Neither true nor false 5: Slightly true 6: Quite true 7: Extremely true**  If you cannot answer one of the items because you have never seen the child in that situation, for example, if the statement is about the child's reaction to your singing and you have never sung to your child, then (√) NA (not applicable). Please be sure to tick (√) a number or NA for every item. | | | | | | | | | |
| --- | --- | --- | --- | --- | --- | --- | --- | --- | --- |
| **No** | **Statement** | **1** | **2** | **3** | **4** | **5** | **6** | **7** | **NA** |
| **My child:** | | | | | | |  |  |  |
| B1. | Seems always in a big hurry to get from one place to another. |  |  |  |  |  |  |  |  |
| B2. | Gets quite frustrated when prevented from doing something s/he wants to do. |  |  |  |  |  |  |  |  |
| B3. | When drawing or colouring in a book, shows strong concentration. |  |  |  |  |  |  |  |  |
| B4. | Likes going down high slides or other adventurous activities. |  |  |  |  |  |  |  |  |
| B5. | Is quite upset by a little cut or bruise. |  |  |  |  |  |  |  |  |
| B6. | Prepares for trips and outings by planning things s/he will need. |  |  |  |  |  |  |  |  |
| B7. | Often rushes into new situations. |  |  |  |  |  |  |  |  |
| B8. | Tends to become sad if the family's plans don't work out. |  |  |  |  |  |  |  |  |
| B9. | Likes being sung to. |  |  |  |  |  |  |  |  |
| B10 | Seems to be at ease with almost any person. |  |  |  |  |  |  |  |  |
| B11. | Is afraid of burglars or the "boogie man." |  |  |  |  |  |  |  |  |
| B12. | Notices it when parents are wearing new clothing. |  |  |  |  |  |  |  |  |
| B13. | Prefers quiet activities to active games. |  |  |  |  |  |  |  |  |
| B14. | When angry about something, s/he tends to stay upset for ten minutes or longer |  |  |  |  |  |  |  |  |
| B15. | When building or putting something together, becomes very involved in what s/he is doing, and works for long periods. |  |  |  |  |  |  |  |  |
| B16. | Likes to go high and fast when pushed on a swing. |  |  |  |  |  |  |  |  |
| B17. | Seems to feel depressed when unable to accomplish some task. |  |  |  |  |  |  |  |  |
| B18. | Is good at following instructions. |  |  |  |  |  |  |  |  |
| B19. | Takes a long time in approaching new situations. |  |  |  |  |  |  |  |  |
| B20. | Hardly ever complains when ill with a cold. |  |  |  |  |  |  |  |  |
| B21. | Likes the sound of words, such as nursery rhymes. |  |  |  |  |  |  |  |  |
| B22. | Is sometimes shy even around people s/he has known a long time. |  |  |  |  |  |  |  |  |
| B23. | Is very difficult to soothe when s/he has become upset. |  |  |  |  |  |  |  |  |
| B24. | Is quickly aware of some new item in the living room. |  |  |  |  |  |  |  |  |
| B25. | Is full of energy, even in the evening |  |  |  |  |  |  |  |  |
| B26. | Is not afraid of the dark. |  |  |  |  |  |  |  |  |
| B27. | Sometimes becomes absorbed in a picture book and looks at it for a long time. |  |  |  |  |  |  |  |  |
| B28. | Likes rough and rowdy games. |  |  |  |  |  |  |  |  |
| B29. | Is not very upset at minor cuts or bruises. |  |  |  |  |  |  |  |  |
| B30. | Approaches places s/he has been told are dangerous slowly and cautiously. |  |  |  |  |  |  |  |  |
| B31. | Is slow and unhurried in deciding what to do next. |  |  |  |  |  |  |  |  |
| B32. | Gets angry when s/he can't find something s/he wants to play with. |  |  |  |  |  |  |  |  |
| B33. | Enjoys gentle rhythmic activities such as rocking or swaying. |  |  |  |  |  |  |  |  |
| B34. | Sometimes turns away shyly from new acquaintances. |  |  |  |  |  |  |  |  |
| B35. | Becomes upset when loved relatives or friends are getting ready to leave following a visit. |  |  |  |  |  |  |  |  |
| B36. | Comments when a parent has changed his/her appearance. |  |  |  |  |  |  |  |  |

1. **PARENTING STYLE**

| Instruction: Answer all questions. For each statement tick (√) at the best description of your parenting style. | | | | | | |
| --- | --- | --- | --- | --- | --- | --- |
| **No** | **Statement** | **Strongly agree** | **Agree** | **Unsure** | **Disagree** | **Strongly disagree** |
| C1 | When I ask my children to do something I expect it to be done immediately without questions. |  |  |  |  |  |
| C2 | Once family rules have been made, I discuss the reasons for the rules with my children. |  |  |  |  |  |
| C3 | I always encourage discussion when my children feel family rules and restrictions are unfair |  |  |  |  |  |
| C4 | Children need to be free to make their own decisions about activities, even if this disagrees with what a parent might want to do |  |  |  |  |  |
| C5 | I do not allow my children to question the decision that I make |  |  |  |  |  |
| C6 | I direct the activities and decisions of my children by talking with them and using rewards and punishment |  |  |  |  |  |
| C7 | Other parents should use more force to get their children to behave |  |  |  |  |  |
| C8 | My children do not need to obey rules simply because people in authority have told them to |  |  |  |  |  |
| C9 | My children know what I expect from them, but feel free to talk with me if they feel my expectations are unfair |  |  |  |  |  |
| C10 | Smart parents should teach their children early exactly who is the boss in the family |  |  |  |  |  |
| C11 | I usually don’t set firm guidelines for my children’s behaviour |  |  |  |  |  |
| C12 | Most of the time I do what my children want when making family decisions |  |  |  |  |  |
| C13 | I tell my children what they should do, but I explain why I want them to do it |  |  |  |  |  |
| C14 | I get very upset if my children try to disagree with me |  |  |  |  |  |
| C15 | I let my children know what behaviour is expected and if they don’t follow the rules they get punished |  |  |  |  |  |
| C16 | I allow my children to decide most things for themselves without a lot of help from me |  |  |  |  |  |
| Instruction: Answer all questions. For each statement tick (√) at the best description of your parenting style. | | | | | | |
|  |  | **Strongly agree** | **Agree** | **Unsure** | **Disagree** | **Strongly disagree** |
| C17 | I listen to my children when making decisions, but I do not decide something simply because my children want it |  |  |  |  |  |
| C18 | I do not think of myself as responsible for telling my children what to do |  |  |  |  |  |
| C19 | I have clear standards of behaviour for my children, but I am willing to change these standards to meet the needs of the child |  |  |  |  |  |
| C20 | I expect my children to follow my directions, but I am always willing to listen to their concerns and discuss the rules with them |  |  |  |  |  |
| C21 | I allow my children to form their own opinions about family matters and let them make their own decisions about those matters |  |  |  |  |  |
| C22 | Most problem in society could be solved if parents were stricter when their child disobey |  |  |  |  |  |
| C23 | I often tell my children exactly what I want them to do and how I expect them to do it |  |  |  |  |  |
| C24 | I set firm guidelines for my children but am understanding when they disagree with me |  |  |  |  |  |
| C25 | I do not direct the behaviours, activities or desires of my children |  |  |  |  |  |
| C26 | My children knows what I expect of them and do what is asked simply out of respect for my authority |  |  |  |  |  |
| C27 | If I make a decision that hurts my children, I am willing to admit that I made a mistake |  |  |  |  |  |

1. **PARENT’S PERCEPTION TOWARDS HOME INJURY PREVENTION**

Instruction: Answer all the questions by ticking (√) at the most relevant answer.

**i. Perceived vulnerability**

| **No** | **Items** | **Strongly agree** | **Agree** | **Unsure** | **Disagree** | **Strongly disagree** |
| --- | --- | --- | --- | --- | --- | --- |
| My child is likely to be injured by: | |  |  |  |  |  |
| D1. | falls from stairs |  |  |  |  |  |
| D2. | burns from hot water/food |  |  |  |  |  |
| D3. | burns from clothing iron |  |  |  |  |  |
| D4. | bums from stove or oven |  |  |  |  |  |
| D5. | burns from firecrackers |  |  |  |  |  |
| D6. | electric shock |  |  |  |  |  |
| D7. | poisoning |  |  |  |  |  |
| D8. | getting a cord or string tightened around his/her neck |  |  |  |  |  |
| D9. | smothering |  |  |  |  |  |
| D10. | cuts from knives and other sharp objects |  |  |  |  |  |

**ii. Perceived severity**

| **No** | **Items** | **Strongly agree** | **Agree** | **Unsure** | **Disagree** | **Strongly disagree** |
| --- | --- | --- | --- | --- | --- | --- |
| (insert injury) would be serious in my child: | |  |  |  |  |  |
| D11. | falls from stairs |  |  |  |  |  |
| D12. | burns from hot water/food |  |  |  |  |  |
| D13. | burns from clothing iron |  |  |  |  |  |
| D14. | bums from stove or oven |  |  |  |  |  |
| D15. | burns from firecrackers |  |  |  |  |  |
| D16. | electric shock |  |  |  |  |  |
| D17. | poisoning |  |  |  |  |  |
| D18. | getting a cord or string tightened around his/her neck |  |  |  |  |  |
| D19. | smothering |  |  |  |  |  |
| D20. | cuts from knives and other sharp objects |  |  |  |  |  |

**iii. Perceived benefit**

| **No** | **Items** | **Strongly agree** | **Agree** | **Unsure** | **Disagree** | **Strongly disagree** |
| --- | --- | --- | --- | --- | --- | --- |
| If I do things to prevent injuries to my child… | |  |  |  |  |  |
| D21. | I don’t worry as much about him/her being injured |  |  |  |  |  |
| D22. | he/she will be less likely to have an injury. |  |  |  |  |  |
| D23. | I will save money on medical bills. |  |  |  |  |  |
| D24. | will save time later. |  |  |  |  |  |
| D25. | suffering to my child can be prevented. |  |  |  |  |  |

**iv. Perceived Barriers**

| **No** | **Items** | **Strongly agree** | **Agree** | **Unsure** | **Disagree** | **Strongly disagree** |
| --- | --- | --- | --- | --- | --- | --- |
| Preventing injuries to my child… | |  |  |  |  |  |
| D26. | is hard to remember. |  |  |  |  |  |
| D27. | is burdensome as I do not have enough money. |  |  |  |  |  |
| D28. | would take too much time. |  |  |  |  |  |
| D29. | is hard because I do not know enough about how to prevent injury to my child. |  |  |  |  |  |
| D30. | is hard because usually I am too tired to do it. |  |  |  |  |  |
| D31. | is too hard. |  |  |  |  |  |
| D32. | is hard because my child will not do as what I say. |  |  |  |  |  |
| D33. | is hard because I do not know how to contact people in the housing authority who will repair the safety hazards in my home. |  |  |  |  |  |
| D34. | is hard because there is too much confusion in my house. |  |  |  |  |  |
| D35. | is hard because my house is too messy. |  |  |  |  |  |
| D36. | is hard because it is difficult for me to get information on how to prevent injuries to my child. |  |  |  |  |  |
| D37. | is hard because there are too many children to take care of. |  |  |  |  |  |

**v. Perceived self-efficacy**

| **No** | **Items** | **Strongly agree** | **Agree** | **Unsure** | **Disagree** | **Strongly disagree** |
| --- | --- | --- | --- | --- | --- | --- |
| I have the ability to.. | |  |  |  |  |  |
| D38. | keep plastic wrappers out of reach of my child. |  |  |  |  |  |
| D39. | keep medicine and household products in a locked cabinet. |  |  |  |  |  |
| D40. | keep knives and sharp objects out of reach of my child. |  |  |  |  |  |
| D41. | have safety caps on all medicine bottles. |  |  |  |  |  |
| D42. | keep matches and cigarette lighters out of reach of my child. |  |  |  |  |  |
| D43. | keep handle of pots and pans when on the stove out of the reach of my child. |  |  |  |  |  |
| D44. | keep electrical appliances and cords out of reach of my child. |  |  |  |  |  |
| D45. | install a toddler safety gate. |  |  |  |  |  |
| D46. | install cabinet locks. |  |  |  |  |  |

1. **SOCIAL NORM**

| Instruction: Answer all the questions by ticking (√) at the most relevant answer. | | | | | | |
| --- | --- | --- | --- | --- | --- | --- |
| **No** | **How much the person below influence you on your home injury prevention?** | **Strong approval** | **Moderate approval** | **Indifferent** | **Moderate disapproval** | **Strong disapproval** |
| E1. | Spouse |  |  |  |  |  |
| E2. | Close relative |  |  |  |  |  |
| E3. | Close friends |  |  |  |  |  |
| E4 | Neighbours |  |  |  |  |  |
| E5. | Medical staffs |  |  |  |  |  |
| E6. | Social welfare officer |  |  |  |  |  |
| E7. | Religious leader |  |  |  |  |  |

1. **PARENT’S ATTITUDE**

| Instruction: All the answers have five choices: Strongly agree, agree, unsure, disagree and strongly disagree. Please tick (√) on the most suitable answer. | | | | | | |
| --- | --- | --- | --- | --- | --- | --- |
| **No** | **Items** | **Strongly agree** | **Agree** | **Unsure** | **Disagree** | **Strongly disagree** |
| F1. | Injuries to children are preventable |  |  |  |  |  |
| F2. | Appropriate supervision by caregivers is one of the most effective methods of injury prevention |  |  |  |  |  |
| F3. | I feel a strong sense of responsibility for the children under my care |  |  |  |  |  |
| F4. | I am in control and feel confident about taking care of the children |  |  |  |  |  |
| F5. | First Aid Training is very useful and important |  |  |  |  |  |
| F6. | Attending workshops and courses on childcare will increase my knowledge on safety and injury prevention |  |  |  |  |  |
| F7. | Whether or not children get injured is largely a matter of fate |  |  |  |  |  |
| F8. | Injuries are helpful because they teach children to handle physical pain better and teach them to be more cautious |  |  |  |  |  |
| F9. | Telling children not to do something will ensure they will not do it |  |  |  |  |  |
| F10. | I think it’s important to let children do whatever they like as long as they are having fun |  |  |  |  |  |
| F11. | Infants do not need as much attention because they cannot walk |  |  |  |  |  |

1. **KNOWLEDGE ON CHILD HOME SAFETY**

| Instruction: All the answers have three choices: Agree, Disagree and Unsure. Please tick (√) on one relevant box. | | | | |
| --- | --- | --- | --- | --- |
| **No** | **Items** | **Agree** | **Disagree** | **Unsure** |
| G1. | I have knowledge on cardiopulmonary resuscitation for children |  |  |  |
| G2. | Children can get a brain hemorrhage just from a fall of low height. |  |  |  |
| G3. | Baby walker is suitable to teach an infant to walk |  |  |  |
| G4. | During the first year of birth, the safest sleeping position for a baby is lying on her back. |  |  |  |
| G5. | Hammock is suitable to put baby to sleep |  |  |  |
| G6. | It is safe for children of 2 years of age to play with coins or any similar object. |  |  |  |
| G7. | A stair gate is needed in a house that is occupied by children younger than 3 years of age. |  |  |  |
| G8. | It is not suitable for a house that occupied by children aged less than 3 years old to have many glass ornaments in their living room |  |  |  |
| G9. | Suitable age for a child to safely bathe alone in the bathroom is 5 years old. |  |  |  |
| G10. | Vitamins are the commonest cause of poisoning in children less than 6 years of age |  |  |  |
| G11. | Children can drown in shallow water |  |  |  |
| G12. | Arrangement of furniture at home can cause injury to the child. |  |  |  |
| G13. | Accidental knock at the edge of a table may cause injury to children’s eye. |  |  |  |
| G14. | It is safe to keep the bleach in an unlocked cabinet under the kitchen sink. |  |  |  |
| G15. | Children can be suffocated while playing with plastic |  |  |  |

1. **PARENT’S HOME INJURY PREVENTION PRACTICE**

| Instruction: Answer all the questions below by ticking (√) on one relevant box. | | | |
| --- | --- | --- | --- |
| **Usage of safety equipment** | | **YES** | **NO** |
| H1. | Use electrical socket cover at any accessible electrical socket. |  |  |
| H2. | Cover sharp edge of the furniture. |  |  |
| **Removal of hazards** | | **YES** | **NO** |
| H3. | Wipe any spillage immediately |  |  |
| H4. | Keep the sharp utensils away from child accessibility. |  |  |
| H5. | Keep the medicine inside a special box away from child accessibility. |  |  |
| H6. | Keep the insecticides/chemicals away from child accessibility. |  |  |
| H7. | Keep the matches at a safe place away from child accessibility. |  |  |
| H8. | Turn the handle of cooking pans far from children’s reach. |  |  |
| H9. | Keep all coins, tie, scarf and belt away from child accessibility. |  |  |
| H10. | Keep the plastic bags away from child accessibility. |  |  |
| H11. | Keep all pails in the toilet emptied |  |  |
| H12. | Ensure the toilet door is closed at all time. |  |  |
| H13. | Ensure to put the child away before handling any hot foods/drinks |  |  |
| H14. | Keep the detergents inside a lock cupboard away from child accessibility. |  |  |
| **Supervise children** | | **YES** | **NO** |
| H15. | Ensure that children are not left alone in the room full of glass decoration. |  |  |
| H16. | Ensure that children do not carry any glassware without adult supervision. |  |  |
| H17. | Ensure that children do not climb any furniture without being noticed. |  |  |
| H18. | Ensure children did not bathe alone in the toilet. |  |  |
| **Teach children on safety measures** | | **YES** | **NO** |
| H19. | Teach children not to put any toys inside their mouth. |  |  |
| H20. | Teach children not to run when going down the stairs. |  |  |
| H21. | Teach children not to wander around while eating. |  |  |

1. **HOUSE HAZARDS**

Instruction: Please tick (√) in the box for hazard that is present in your house and within reach from your child.

| **Risk of injury** | **Hazards** | **Present & within child’s reach** |
| --- | --- | --- |
| **Poisonings by solids and liquids** | Pill medications |  |
|  | Tube medications |  |
|  | Inhaler medications |  |
|  | Liquid medications |  |
|  | Detergents/Cleansers |  |
|  | Polishes/Waxes |  |
|  | Alcoholic beverages |  |
|  | Beauty products |  |
|  | Insecticides/Rodenticides |  |
|  | Paints/Stains |  |
|  | Solvents/Thinners |  |
|  | Glues/Adhesives |  |
|  | Petroleum products |  |
|  | Fertilizers/Herbicides |  |
|  | Poisonous plants |  |
| **Burns and scalds** | Combustibles |  |
|  | Outlets/switches without covers |  |
|  | Frayed electrical cords/plugs |  |
| **Suffocation** | Plastics |  |
|  | Hanging cords/wires |  |
|  | Small objects |  |
| **Cuts** | Sharp objects |  |
| **Fall** | Balconies |  |
|  | Stairs/Steps |  |
|  | Windows without protective devices |  |
| **Drowning** | Bathtubs/sinks |  |
|  | Buckets |  |
|  | Wading pools containing 2 inch of liquid |  |

**THANK YOU FOR YOUR TIME AND HONESTY.**
