# Supplementary material for: Determinants of non-adherence to home injury prevention practice among parents of under-five children in North Seberang Perai district, Penang: A mixed-methods study protocol
Source: PLoS One. 2023 Aug 16;18(8):e0282995. doi: 10.1371/journal.pone.0282995 (PMC10431611; doi:10.1371/journal.pone.0282995)
Supplement: Attachment — Submitted filename: comment to reviewers (dr iman, plos one)_updated 13 jan.pdf [file pone.0282995.s004.pdf]

REPLIES TO COMMENTS OF REVIEWERS

Dear Dr./ Mr./Ms. (Editor),

Thank you for giving me the opportunity to submit a revised draft of my manuscript titled *Determinants of non-adherence to home injury prevention practice among parents of under-five children in North Seberang Perai district, Penang: A mixed methods study protocol* to *PLOS ONE*. I appreciate the time and effort that you and the reviewers have dedicated to providing your valuable feedback on my manuscript. I am grateful to the reviewers for their insightful comments on my paper. I have been able to incorporate changes to reflect most of the suggestions provided by the reviewers. I have highlighted the changes within the manuscript. Here is a point-by-point response to the reviewers' comments and concerns.

Review Comments to the Author

Reviewer	Comments	Reply from Author
1	*Question 1: The rationale and justification to conduct this study are well described and understood*	Thank you for the positive evaluation of our manuscript.
	Question 2: Need some improvements on the objectives arrangement. It is best to put objective 3 (the association between independent variables and low injury practice) as objective no 2 and determinants of low injury practice as no 3.	Thank you for pointing this out. We agree with this comment. Therefore, we have changed the arrangement of the objectives as you have suggested as below:<br>i. To determine the prevalence and type of unintentional home injury among under-five children;<br>ii. To determine the association between the independent variables with low unintentional home injury prevention practice among parents of under-five children in North Seberang Perai District, Penang.<br>iii. To determine the determinants of low unintentional home injury prevention practices among parents of under-five children;
	Question 3: Methodology is feasible but some part did not mention in detail. Need improvement.<br>*Some of the comments that need improvement:*<br>*Methodology for Qualitative phase: It is best if author provides the details of Question/Probing questions that are planned to be asked in this study.*	We agree with this and have incorporated your suggestion throughout the manuscript. We have shared the details of the probing questions for the qualitative phase in appendix.

	Question 4: No comment	
	Question 5: Some errors showed in the reference section. A typo error on Institute of Public Health reference no. 19. References: author needs to review the standard way of writing the references following the format for PLOS. There are few references need to be looked into ie. no 6 (is this from a journal or a book/others), no 9 (is this from journal or other?), no 19, 20, 21 (IPH: standardise the name), no 10 & 26: redundant, no 31. (Capital letter for Journal's name)	Thank you for the comments. Here are the corrected version:<br><br>Reference No 19- Typo error resolved.<br>19. Institute of Public Health (IPH). National health and morbidity survey 2006.pdf.<br><br>Reference No 6: This reference is from a journal.<br>Leblanc JC, Pless IB, King WJ, Bawden H, Klassen T, Tenenbein M. Home safety measures and the risk of unintentional injury among young children: a multicentre case – control study. 2006.<br><br>No 10 and 26 are not the same references.<br><br>Reference No 9: This reference is from a journal.<br>Lao Z, Gifford M, Dalal K. Economic cost of childhood unintentional injuries. *Int J Prev Med*. 2012;3(5):303-312.<br><br>Reference No 19,20,21- Name has been standardised.<br>19. Institute of Public Health (IPH). National health and morbidity survey 2006.pdf.<br>20. Institute of Public Health (IPH). National health and morbidity survey 2011.pdf.<br>21. Institute of Public Health (IPH). National health and morbidity survey 2016 : Maternal And Child Health (MCH). *Kementeri Kesihat Malaysia*. 2016;2:276.<br><br>Reference No 31-Resolved.<br>Russell KM, Champion VL. Health beliefs and social influence in home safety practices of mothers with preschool children. Image: The journal of nursing scholarship. 1996 Mar;28(1):59-64.
Reviewer 2	*Overall comments: This paper requires a significant review from a sentence structure and grammatical perspective. There are several fragmented sentences and areas where words are pluralized when they should not be, or vice versa.*<br><br>*There are several areas where the authors need to provide justification for methodological choices and areas where much more information is needed.*	We thank the reviewer for their constructive and thoughtful comments. The mistakes have been corrected and highlighted in yellow.

	There should also be a section that describes the challenges that may be presented in doing this work and how the authors plan to mitigate these challenges.	
	Introduction: The data presented in the introduction is outdated, please update with more recent data.	We understand the concern of the reviewer. Most of the data used for introduction were dated 2013 and before, showing that there is lacking in recent study or publication on injury. This rings true when even WHO have not updated their World report on child injury prevention which was last published in 2008.
	I appreciate the iceberg analogy – please explain this further in your description for readers that may not be familiar.	We thank the reviewer for the appreciation. We agree that not all readers are familiar with this iceberg analogy. We already added some explanation about it in the manuscript and highlighted in yellow.<br><br>UNICEF describes the burden of injury similar to the iceberg phenomenon refers to a situation where what we see is just a small portion of the actual size of the problem. In this case, most of child home injury are hidden from the public because they are unreported.
	You should spell out TB and HIV.	The corrected version is below. We have, replaced the abbreviation to full spelling throughout the manuscript to ensure understanding of the readers especially who are not in health and medical background.<br><br>The burden of injury is almost two times higher than of Tuberculosis and Human Immunodeficiency Virus.
	You need to review your paper for tense. Some areas speak in present and others in past tense. Should be consistent.	As suggested by the reviewer, we have revised the tense used in the manuscript and make it consistent in the whole manuscript.
	Is there a way to have another review of this paper to revise for sentence structure? I am not sure if it is a non-English as a first language issues, but it would help readers to do another review for clarity. For example: In Malaysia, injury mortality among under-five was found to be static since 1990 until 2013 despite a	We have followed the recommended suggestion.

	marked reduction in death from the communicable disease [4]. Eight percent of under-five mortality in Malaysia is contributed by injury and the leading cause of death is drowning followed by road traffic accident, burn, choking and poisoning [5].<br>*This would be clearer if written this way: In Malaysia, injury mortality for children under the age of 5 years remained unchanged from the years 1990 to 2013 inclusive, despite a marked reduction in death from all communicable diseases. Further, 8% of all mortality in children under the age of 5 years is caused by injury, with drowning, followed by road traffic collisions, burns, choking and poisonings as the leading causes of death.*<br>*There are several areas where words are pluralized, and shouldn't be.*	
	Page 3: Injury incidence was found to be significantly associated with poor prevention practice [10].<br>*Is there a context to this sentence? Injuries in the home specifically?*	We thank the reviewer for pointing out the confusion. The injury incidence refers to injuries at home among children, while, poor prevention practice refers to parental prevention practice on their child's safety. We have modified the sentence based on your suggestion for more clarity and highlighted in yellow.<br><br>Injury incidence at home was found to be significantly associated with poor parental prevention practice on their child's safety.
	Page 4: A local study also shows similar findings where only five to eleven percent of children were found to wear their seatbelts while riding cars and only around 30 percent of children were	The statement is deleted from paragraph as it is not related to home injury.

	found wearing helmets while riding motorbikes [11].<br>*Several issues here: First, you are making the point about injuries in the home and this study does not support that, further, children are reported to be riding motorcycles? I would imagine this is as passengers? Please clarify.*	
	Page 4: Most studies are quantitative and focus on injury rate rather than its prevention.<br>*This sentence is misleading. Quantitative studies contribute to the breadth of knowledge for prevention including, but not limited to randomised controlled trials that evaluate the effectiveness of prevention interventions. Further, given you are using a mixed methods approach, you should justify the use of both methods. What research design are you using for the “quantitative” study?*	We thank the reviewer for their constructive and thoughtful comments. We have mentioned the research design for the quantitative study in the manuscript.<br><br>This quantitative research uses analytical cross-sectional (observation) design where the data is collected at one point of time without introducing any intervention to the participants. The analysis will include univariate (Chi Square or Fisher's Exact) and bivariate (Multiple Logistic Regression).<br><br>What I meant from the sentence "Most studies are quantitative and focus on injury rate rather than its prevention" is from my literature review, most studies directly measure the prevalence of injury but neglected to find the prevalence of injury prevention which what this study plans to do. Quantitative study has its limitation where a researcher cannot explore something that is not already in the study tool or scale. Quantitative study cannot help researcher understand the in-depth reason behind a behavior, in this case non-adherence to child home injury prevention, where naturally we would think all parents want their children to be safe. Thus in doing so, qualitative study is warranted as from our literature review, there are limited qualitative study on this matter in Malaysia.
	It is unclear why you chose the inclusion criteria that you have, please explain. What selection bias is inherent when you chose families that visit maternal child health clinics?	The inclusion criteria for this study is:<br>i. Parent of a child age less than five years old. If there are more than one child age below five, the younger will be chosen as the studied child. This is because children below five years old are at the most risk of sustaining fatal home injury. The youngest of the siblings are chosen as studies have shown that parents will be focusing and carrying out most prevention on the youngest as they would think they are the most susceptible. (Al-Khameesa, 2006; İnce et al., 2017)<br>ii. Parent age 18 and above, - adults are chosen as they are considered well developed mentally, physically and socially to take care of children.<br>iii.Parent is a Malaysian citizen who can provide his/her own valid identification card. - we only choose Malaysian citizen because the income and social gap between Malaysians and non-Malaysian is quite big which may influence many factors including knowledge

		and social norm. This might cause outlier in the study findings later on.<br><br>The study cite (Maternal Health Clinic) will be chosen as the clinic could provide a list of parents with under five-aged children for sampling purpose. It is not feasible to get a sampling frame from a community-based registry of parents with under five children.
	There needs to be more information on your sample size calculation.	The information on sample size calculation has been added to the manuscript.<br><br>Sample size estimation for this study was calculated using two group proportion formula by Hosmer & Lemeshaw (1990). Prior data by Honda & Nagata (2019) that look into the associated factors of injury prevention practice indicated that the proportion of the parents with poor adherence to injury prevention practice with index male index child is 0.416 and the proportion of the parents with poor adherence to injury prevention practice with female index child is 0.276.<br>$n = \frac{\left(z_{1-\alpha}\sqrt{2\bar{P}(1-\bar{P})} + z_{1-\beta}\sqrt{P_1(1-P_1)+P_2(1-P_2)} \right)^2}{(P_1-P_2)^2}$<br>n = sample size estimation<br>$z(_{1-\alpha})$ = Standard error associated with 95% confidence interval = 1.96<br>$z(_{1-\beta})$ = Standard error associated with 80% power = 0.842<br>P_1 = population proportion 1<br>P_2 = population proportion 2<br>Ṕ = $(P_1+P_2)/2$<br>P_1 = 0.416 (Proportion of poor adherence parents with male index child)<br>P_2 = 0.276 (Proportion of poor adherence parents with female index child)<br>Ṕ = $(P_1+P_2)/2$ = (0.416 + 0.276) / 2 = 0.346<br><br>n = {1.96 √2(0.346)[1-(0.346)] + [0.842 √ (0.416)(1-0.416) + (0.276)(1-0.276)]}^{2}<br>(0.416 - 0.276)^2<br>n = 181.21<br><br>The minimum sample size per group is 181.<br>There will be two groups of child age in this study, therefore 181 x 2 = 362<br>For this study 20% drop out is used for sample size adjustment.<br>Adjust for 20% drop out: 362 / 80% = 452.5 → rounded to 453.<br>Therefore, the sample size for this study is 453.
	Page 9: Every 3rd patient (a random number generator generates number 3) from the list will be	We use systematic random sampling method. We already explained the details in the manuscript and highlighted in yellow.

	selected.<br>*This is actually not random, please explain the use of this method.*	In this research, we use systematic random sampling method where individuals are selected at regular intervals from the sampling frame. The sampling frame will be an appointment list where not only under-five children but all patients under The Maternal Child Health unit will be listed too. From the daily list, we will exclude the non-under-five patients, and then from the list we will choose every third to be approached. Number 3 is chosen because during pandemic time, number of patients reduced markedly so if we choose a bigger number, we might not have enough patient for a day data collection as clinic only run from 8am to 5pm weekdays.
	Page 11: The score will be calculated by summing the individual items in the scale. The items in bold need to be recoded in reverse before summing. Please explain what this means.	The corrected statement is as below:<br><br>Reverse scoring means that the numerical scoring scale runs in the opposite direction. The behavioural tendencies of the child are rated by the parent on a seven-point scale, ranging from 'Never' (value of 1) to 'Always' (value of 7).<br>So, in reverse scoring, 'Never' would give a score of 7, 'Very Rarely' would be 6, 'Less than half the time' equals to 5, 'About half the time' stills 4, 'More than half the time' equals to 3, 'Almost always' equals to 2, 'Always' will be 1.<br>This is done based on the respondent's answer in the questionnaire. Summing means to add all the individual item Likert scale to become a total score.
	Page 14: Your data analyses section is quite short. Please provide more explanation to how you are modeling the data, what variables you will use in your model, how the data will be presented.<br><br>*For the qualitative analyses, how will you present these data? By high level theme only? How will the data be categorized?*	More explanation has been added in data analyses section.<br>The data will be presented according to the level of analysis and into separated table: Parental characteristics, child characteristics and environmental characteristics. Firstly, the descriptive data will be presented in frequency and percentage. Then, Chi Square/Fisher Exact test results will be presenting the n, %, X2 and p-values according to categories in each variable. Lastly, a table of multiple logistic regression will be presented with Beta coefficient, standard error, odds ratio, 95% confidence interval and p-value will be presented. For demographic characteristics, the categorization comes from the operational definitions that were derived from the literature.<br><br>This issue is already stated in the manuscript under data analysis procedure. The qualitative data will be presented in this order: theme - subtheme - codes. The header will be the main theme, then the subthemes and coding will be presented with an appropriate verbatim quotes to illustrate the findings.<br><br>Thematic analysis will be carried out by two researchers who are experienced in thematic analysis process. The steps consist of familiarization, data coding, identifying themes, reviewing themes, refining themes and producing report. At first, familiarization will

		be done by re-reading each transcript to be immersed and acquainted with the data. The second step involves initial data coding to identify the key features of the interview. The interview text will be organized by labelling different words or phrase or sentences into certain categories. Next, we will identify the themes by combining the codes into themes scrutinizing the codes to identify significant one, but broader patterns of themes. The fourth step is to review the themes by comparing the themes that emerged in each transcript to determine whether these data could answer the research questions. In this regard, the themes should be meaningfully connected to the codes identified earlier and should be differentiable. The next step is to refine the themes, which involved defining, naming, and finalizing the themes. This process includes a comprehensive analysis of each theme which focuses on the deeper meaning behind each theme. The last step is to produce the report where all the analysis will be used with supporting verbatim for each theme. The report will consist of a result of analysis of the verbatim, themes that come out from the analysis and the discussion of the themes. An unexpected result that does not belong to any theme may also be included in the report.
	Page 15: The sampling will be stopped when the saturation point is achieved.<br>*Please explain in more detail how you determine saturation.*	We have added an explanation about this saturation point in the manuscript and highlighted in yellow.<br><br>Saturation point is achieved when no more new information is obtained after multiple interviews. Data saturation is reached when there is enough information to replicate the study when the ability to obtain additional new information has been attained, and when further coding is no longer feasible.
	Page 15: A semi-structured interview will be used in this study with the help of an interview guide. The interview guide is prepared with the guidance of a few qualitative experts and previous literatures. Please provide the interview guide as a table or supplementary table. It will be quite key to see how you are asking these questions, given you are a priori selected those participants that scored "poor practice". Further to this point, there needs to be more information on how you are selecting	We thank the reviewer for the great comment. As this is a sequential explanatory mixed method, the interview guide will be developed after the quantitative result is obtained. The significant variables will be used to choose the qualitative participants. For example, the significant variables are younger parents, low education level, child with negative affectivity temperament and poor knowledge, then we will choose parents that fulfil all of these categories to be interviewed.<br><br>We have included the interview guide as appendix in different document than the manuscript.

	the parents for the interviews. Are they based on one set of scores or a combination of several scores?	
	Page 15: There are several areas where you mention examining for face and content validity, there needs to be much more information on how you plan to do this. There should also be suggestions or plans for this if the questions require revision.	As suggested by the reviewer, we have attempted to explain more about the validity in the manuscript. The additional information was highlighted in yellow as below.<br><br>Face validity will be carried out with five parents from the same population but are not included in the study. One parent will be approached where all of their incomprehension or confusion will be addressed until they can fully understand the whole questionnaire. They will provide feedback on their understanding of the items in the questionnaire. Corrections will be done in response to the comment, and the process repeats until all items are cleared. This process will be repeated with multiple parents (one at a time) until there is no more issue is raised.<br><br>Meanwhile, for content validity, subject matter experts (SMEs) from different fields will be asked to review the contents of the questionnaires. For content validity, we plan to approach eight subject matter experts (SMEs) from various expertise. They include a public health specialist, a family medicine specialist, an injury specialist and a paediatrician. The feedback will be in the form of whether the item is essential, useful, but not essential or not necessary for the research. They will be looking into the clarity, accuracy, language and cultural relevance of every item. The feedback will be used to measure content validity ratio (CVR) with the calculation described by Ayre & Scally (2014) [38]. They will be given the questionnaire, at the end of each item there is a box for them to rate as 1 to 3, 1: not necessary, 2: useful but not essential and 3: essential. Next to the box is another box for them to write their comment if any. For each item, CVR is calculated with the formula CVR = (n - N/2)/(N/2), where n is the number of SMEs who describe an item as essential and N is the total number of SMEs involved. Only items with CVR more than 0.9 will be accepted.
	Page 15: Please describe more on how the data from the "pilot" will be used. What possible outcomes could occur here? Would you change the questions? Potentially change your sampling frame?	As suggested by the reviewer, we have added more explanation on this issue and highlighted in yellow.<br><br>The purpose of the Pilot Test is to evaluate the feasibility of sample size, time, cost, risk, and performance of our research project. The pilot study will be carried out with the interview guide prepared and researcher will note any issues developed during the interview and address it by making changes to the interview setting, interview guide, ways of conveying questions and responses etc. When no more issue rises during the interview, the actual study can start.

		Moreover, by getting the result from the pilot test, items that show Cronbach's Alpha value below 0.7 will be deleted from the questionnaire in order to achieve better internal consistency.
	Page 17: …First during the first phase where the quantitative results help plan the follow-up qualitative data collection and the second integration when the results of the quantitative study are connected with the results of the qualitative study to derive a conclusion whether the qualitative results can explain the quantitative results.<br><br>*Please explain this in a different way – do you mean that your quantitative results will inform your qualitative data collection? And then you will use the results of both to provide a more comprehensive discussion of the results?*<br><br>*It is unclear what you mean by follow up qualitative data collection, you are collecting these data twice?*<br><br>*Also, please explain how you will ensure the validity of the data collected from the interviews.*	Integration of quantitative and qualitative data refers to mixing both data as a finding. We have added the explanation regarding this matter in the manuscript.<br><br>This integration will occur when the quantitative results will be used as the inclusion criteria of qualitative participants and acts as a guidance in developing the interview guide. The second data integration will occur when qualitative data is able to explain the quantitative results, for example if parental knowledge is significant variable (in quantitative research), the interview during qualitative research should focus on how much you ever heard or learn about home injury prevention.<br><br>The follow-up in the sentence means that the quantitative data is collected first, followed by qualitative data collection, as it is a sequential mixed methods design.<br><br>We have added the explanation on validity in the manuscript as your suggestion and it was highlighted in yellow.<br><br>In qualitative research, the data needs to be rigour which reflects the trustworthiness and truthfulness, this is based on the researcher's credibility, transferability, dependability and confirmability (Korstjens & Moser, 2018).<br>Credibility assures those research findings represent the credible information gathered from the novel data from the interviewees and provides a real interpretation of the interviewees' real views. For this study, credibility will be ensured through 'member check' process (Morse, Barrett, Mayan, Olson & Spiers, 2002). Once the transcripts of the interview are ready, it will be read to the interviewee to check whether they agree with the content. The correction will be made based on the interviewee's comment in

		order to avoid false interpretation. Peer review also needs to be done to check whether the findings suit the qualitative data. Transferability of this study is ensured by detailing all the research process from study location, sampling population, sampling methods, interview questions and so on to help other researchers to evaluate whether the study is applicable at another setting. Similar to reliability for a quantitative study, dependability shows the extent to which the research would produce similar findings if it is to be repeated. To ensure this quality, this study provides a detailed methodological information of the study, which will help other researchers to carry out a similar study in their setting. Audit trail; a detailed explanation of the data collection and data analysis process will be prepared.<br>The confirmability of this study will be ensured by taking detailed field notes during interview. After the interview was transcribed, the researcher needs to listen to the recording to verify the transcription's accuracy.
	Discussion and conclusion: The authors should describe any challenges that may be presented in doing this work and how they plan to mitigate these challenges.	We thank the reviewer for the valuable suggestion regarding our discussion and conclusion. As suggested by the reviewer, we have added our challenges in doing this research and their solution in our manuscript and were highlighted in yellow.<br><br>Challenges<br>The challenge that might be faced during this study is the COVID-19 pandemic situation where the researcher needs to meet a lot of participants and spends a lot of time with them especially for interviews, making the researcher at risk of contracting COVID-19. Therefore, it is important to wear PPE such as facemask and face shield and observe good SOP at all time. The clinics will have reduced patients as they do need to reduce the crowd. Some clinics even reduce the appointment to only half day. This will cause lack of patients to be approached in a day, which may prolong the time to achieve the adequate sample size. Therefore, we had to sample a bit frequent where every third from the sampling frame in order to complete the study in the planned time. Moreover, health clinic-based studies can introduce some bias including selection bias as there is certain demography that is most likely to attend government health clinics. This will be addressed by doing an analysis with standardized level of income.<br><br>Limitation<br>Another limitation that arrives with pandemic is during the in-depth interview process. As participants need to wear mask during interview, it will be a challenge to observe their facial expression that is vital in qualitative data analysis. This limitation will hopefully be supported by participant's intonation and body language that can still be visible during the interview.
Reviewer 3	*This is a study protocol of a mixed method study. I suggest to review the information on the*	We thank the reviewer for the fair evaluation of our manuscript. As suggested by the reviewer, we have revised the information in the discussions for improvement. Any additional information is highlighted in yellow.

	discussions so that this study protocol is reproducible and can help others to conduct the study of same interest.	
