# Supplementary material for: Determinants of non-adherence to home injury prevention practice among parents of under-five children in North Seberang Perai district, Penang: A mixed-methods study protocol
Source: PLoS One. 2023 Aug 16;18(8):e0282995. doi: 10.1371/journal.pone.0282995 (PMC10431611; doi:10.1371/journal.pone.0282995)
Supplement: Attachment — Submitted filename: PONE-D-20-40899.docx [file pone.0282995.s003.docx]

PONE-D-20-40899

Overall comments: This paper requires a significant review from a sentence structure and grammatical perspective. There are several fragmented sentences and areas where words are pluralized when they should not be, or vice versa. There are several areas where the authors need to provide justification for methodological choices and areas where much more information is needed. There should also be a section that describes the challenges that may be presented in doing this work and how the authors plan to mitigate these challenges.

Specific comments:

Introduction:

The data presented in the in the introduction is outdated, please update with more recent data.

I appreciate the iceberg analogy – please explain this further in your description for readers that may not be familiar.

You should spell out TB and HIV.

You need to review your paper for tense. Some areas speak in present and others in past tense. Should be consistent.

Is there a way to have another review of this paper to revise for sentence structure? I am not sure if it is a non-English as a first language issues, but it would help readers to do another review for clarity. For example: In Malaysia, injury mortality among under-five was found to be static since 1990 until 2013 despite a marked reduction in death from the communicable disease [4]. Eight percent of under-five mortality in Malaysia is contributed by injury and the leading cause

of death is drowning followed by road traffic accident, burn, choking and poisoning [5].

This would be clearer if written this way: In Malaysia, injury mortality for children under the age of 5 years remained unchanged from the years 1990 to 2013 inclusive, despite a marked reduction in death from all communicable diseases. Further, 8% of all mortality in children under the age of 5 years is caused by injury, with drowning, followed by road traffic collisions, burns, choking and poisonings as the leading causes of death.

There are several areas where words are pluralized, and shouldn’t be.

Page 3: Injury incidence was found to be significantly associated with poor prevention practice [10].

Is there a context to this sentence? Injuries in the home specifically?

Page 4: A local study also shows similar findings where only five to eleven percent of children were found to wear their seatbelts while riding cars and only around 30 percent of children were

found wearing helmets while riding motorbikes [11].

Several issues here: First, you are making the point about injuries in the home and this study does not support that, further, children are reported to be riding motorcycles? I would imagine this is as passengers? Please clarify.

Page 4: Based on the National Health and Morbidity Survey 2006, 2011 and 2016, the prevalence of child injury is highest in a high income family [19][20][21]. The contradicting finding is unique to Malaysia, and as to our knowledge, has never been explored yet.

Please clarify what type of injury you mean here, all injury? All unintentional injury?

Page 4: Most studies are quantitative and focus on injury rate rather than its prevention.

This sentence is misleading. Quantitative studies contribute to the breadth of knowledge for prevention including, but not limited to randomised controlled trials that evaluate the effectiveness of prevention interventions. Further, given you are using a mixed methods approach, you should justify the use of both methods. What research design are you using for the “quantitative” study?

It is unclear why you chose the inclusion criteria that you have, please explain. What selection bias is inherent when you chose families that visit maternal child health clinics?

There needs to be more information on your sample size calculation.

Page 9: Every 3rd patient (a random number generator generates number 3) from the list will be selected.

This is actually not random, please explain the use of this method.

Page 11: The score will be calculated by summing the individual items in the scale. The items in bold need to be recoded in reverse before summing.

Please explain what this means.

Page 14: Your data analyses section is quite short. Please provide more explanation to how you are modeling the data, what variables you will use in your model, how the data will be presented. For the qualitative analyses, how will you present these data? By high level theme only? How will the data be categorized?

Page 15: The sampling will be stopped when the saturation point is achieved.

Please explain in more detail how you determine saturation.

Page 15: A semi-structured interview will be used in this study with the help of an interview guide. The interview guide is prepared with the guidance of a few qualitative experts and previous literatures.

Please provide the interview guide as a table or supplementary table. It will be quite key to see how you are asking these questions, given you are a priori selected those participants that scored “poor practice”. Further to this point, there needs to be more information on how you are selecting the parents for the interviews. Are they based on one set of scores or a combination of several scores?

Page 15: There are several areas where you mention examining for face and content validity, there needs to be much more information on how you plan to do this. There should also be suggestions or plans for this if the questions require revision.

Page 15: Please describe more on how the data from the “pilot” will be used. What possible outcomes could occur here? Would you change the questions? Potentially change your sampling frame?

Page 17: …First during the first phase where the quantitative results help plan the follow-up qualitative data collection and the second integration when the results of the quantitative study are connected with the results of the qualitative study to derive a conclusion whether the qualitative results can explain the quantitative results.

Please explain this in a different way – do you mean that your quantitative results will inform your qualitative data collection? And then you will use the results of both to provide a more comprehensive discussion of the results? It is unclear what you mean by follow up qualitative data collection, you are collecting these data twice? Also, please explain how you will ensure the validity of the data collected from the interviews.

Discussion and conclusion: The authors should describe any challenges that may be presented in doing this work and how they plan to mitigate these challenges.
