# Supplementary material for: Determinants of non-adherence to home injury prevention practice among parents of under-five children in North Seberang Perai district, Penang: A mixed-methods study protocol
Source: PLoS One. 2023 Aug 16;18(8):e0282995. doi: 10.1371/journal.pone.0282995 (PMC10431611; doi:10.1371/journal.pone.0282995)
Supplement: Attachment — Submitted filename: PONE-D-20-40899_R2.docx [file pone.0282995.s005.docx]

| ***Review 1*** | **Response** | **Review 2** |
| --- | --- | --- |
| *Introduction: The data presented in the introduction is outdated, please update with more recent data.* | We understand the concern of the reviewer. Most of the data used for introduction were dated 2013 and before, showing that there is lacking in recent study or publication on injury. This rings true when even WHO have not updated their World report on child injury prevention which was last published in 2008. | There is more updated literature in the area of at home injury prevention for children and youth, including a 2019 systematic review: https://www.ncbi.nlm.nih.gov/pmc/articles/PMC7137865/. |
| *Is there a way to have another review of this paper to revise for sentence structure? I am not sure if it is a non-English as a first language issues, but it would help readers to do another review for clarity.* | We have followed the recommended suggestion. | There is no indication in the revised paper where the changes were made. As a reviewer, it is very helpful to do the second review seeing where changes were made.  In reading the manuscript again, I can see that some changes were made; however, I still believe that this is not at the level for publication. |
| *Most studies are quantitative and focus on injury rate rather than its prevention. This sentence is misleading. Quantitative studies contribute to the breadth of knowledge for prevention including, but not limited to randomised controlled trials that evaluate the effectiveness of prevention interventions. Further, given you are using a mixed methods approach, you should justify the use of both methods. What research design are you using for the “quantitative” study?* | What I meant from the sentence "Most studies are quantitative and focus on injury rate rather than its prevention" is from my literature review, most studies directly measure the prevalence of injury but neglected to find the prevalence of injury prevention which what this study plans to do. Quantitative study has its limitation where a researcher cannot explore something that is not already in the study tool or scale. Quantitative study cannot help researcher understand the in-depth reason behind a behavior, in this case non-adherence to child home injury prevention, where naturally we would think all parents want their children to be safe. Thus in doing so, qualitative study is warranted as from our literature review, there are limited qualitative study on this matter in Malaysia. | This response is very unclear. I am unsure how to interpret this response; therefore, this remains unanswered. |
| *What selection bias is inherent when you chose families that visit maternal child health clinics?* | The study cite (Maternal Health Clinic) will be chosen as the clinic could provide a list of parents with under five-aged children for sampling purpose. It is not feasible to get a sampling frame from a community-based registry of parents with under five children. | The authors did not address my question on selection bias. |
| *Discussion and conclusion: The authors should describe any challenges that may be presented in doing this work and how they plan to mitigate these challenges.* | We thank the reviewer for the valuable suggestion regarding our discussion and conclusion. As suggested by the reviewer, we have added our challenges in doing this research and their solution in our manuscript and were highlighted in yellow. | The authors provide one limitation on the topic of COVID-19, and mention selection bias as another. There are no comments on how selection bias would affect the analyses and interpretation of the data.  This section remains insufficient as there are other challenges inherent in the study design that are not discussed. |
