# Supplementary material for: Determinants of non-adherence to home injury prevention practice among parents of under-five children in North Seberang Perai district, Penang: A mixed-methods study protocol
Source: PLoS One. 2023 Aug 16;18(8):e0282995. doi: 10.1371/journal.pone.0282995 (PMC10431611; doi:10.1371/journal.pone.0282995)
Supplement: Attachment — Submitted filename: response to reviewer december 2022 (plos one).docx [file pone.0282995.s006.docx]

| **Review** | **Response** |
| --- | --- |
| Reviewer #1: Few comments regarding writing typo error in the text such as 1) pg 12 bottom line - repetitive of word index  Author needs to get final proofreading for english grammatical order.  Some of the reference were not written correctly following format ie. the WHO report in PDF from website, must have URL, date accessed | The typo error in the text has been corrected. The repeated word has been removed. We have done final proofreading for English grammatical order of the manuscript. |
| Introduction: The data presented in the introduction is outdated, please update with more recent data.  There is more updated literature in the area of at home injury prevention for children and youth, including a 2019 systematic review: https://www.ncbi.nlm.nih.gov/pmc/articles/PMC7137865/. | Thank you for the suggestion. However, the suggested study was an interventional study that introduce interventions such as education and training to improve parental health belief constructs and motivation to conduct preventive measures. It cannot be applied to this study as we measure how parental prevention practice influenced by parental, child and environmental factors. This study has very limited references. |
| Is there a way to have another review of this paper to revise for sentence structure?? I am not sure if it is a non-English as a first language issues, but it would help readers to do another review for clarity.  In reading the manuscript again, I can see that some changes were made; however, I still believe that this is not at the level for publication. | The manuscript has been proofread. |
| Most studies are quantitative and focus on injury rate rather than its prevention. This sentence is misleading. Quantitative studies contribute to the breadth of knowledge for prevention including, but not limited to randomised controlled trials that evaluate the effectiveness of prevention interventions. Further, given you are using a mixed methods approach, you should justify the use of both methods. What research design are you using for the “quantitative” study? | Thank you for the comment and the statement may be misinterpreted. Although the relationship between child injury and its prevention has been established by previous studies, subsequent researchers tend to directly relates injury (outcome) to many variables or associated factors and exclude preventive measures (that may have or have not been taken) by the parent/guardian. Thus, this study is trying to fill that gap in terms of home injury among the Malaysian population.  The quantitative study will be using Cross-sectional design. This is used as we are looking into the association parental home injury prevention practice with many variables which is suitable to be studied using questionnaire at one point of time. The following qualitative study plans to explains the findings from the quantitative study, which can only be answered by the same respondents. This will be achieved by using in-depth interview that is suitable for a sensitive and private issue such as parenting and child injury where parents can be embarassed to discuss in a group (FGD). |
| What selection bias is inherent when you chose families that visit maternal child health clinics? | Selection bias that may occur due to the study location is the sample may consist more of mothers home-makers, lower income and lower education as these clinics' clients are largely made of these demographic groups. |
| Discussion and conclusion: The authors should describe any challenges that may be presented in doing this work and how they plan to mitigate these challenges.  The authors provide one limitation on the topic of COVID-19, and mention selection bias as another. There are no comments on how selection bias would affect the analyses and interpretation of the data.  This section remains insufficient as there are other challenges inherent in the study design that are not discussed. | We thank the reviewer for the valuable suggestion regarding our discussion and conclusion. As suggested by the reviewer, we have added our challenges in doing this research and their solution in our manuscript and were highlighted in yellow. |
